# Antimicrobial Activity, Microstructure, Mechanical, and Barrier Properties of Cassava Starch Composite Films Supplemented With Geranium Essential Oil

**DOI:** 10.3389/fnut.2022.882742

**Published:** 2022-05-11

**Authors:** Bin Wang, Shouxin Yan, Lizhong Qiu, Wei Gao, Xuemin Kang, Bin Yu, Pengfei Liu, Bo Cui, A. M. Abd El-Aty

**Affiliations:** ^1^State Key Laboratory of Biobased Material and Green Papermaking, Shandong Academy of Sciences, Qilu University of Technology, Jinan, China; ^2^School of Food Science and Engineering, Qilu University of Technology, Shandong Academy of Sciences, Jinan, China; ^3^Zhucheng Xingmao Corn Developing Co., Ltd., Zhucheng, China; ^4^Department of Pharmacology, Faculty of Veterinary Medicine, Cairo University, Giza, Egypt; ^5^Department of Medical Pharmacology, Medical Faculty, Ataturk University, Erzurum, Turkey

**Keywords:** cassava starch-based films, physicochemical properties, antibacterial effect, packaging biomaterial, food packaging

## Abstract

In this study, we prepared cassava starch-based films by the casting method. Afterwards, the effects of geranium essential oil (GEO) on the prepared films' physicochemical, morphology, and antibacterial properties were revealed. We found that the thickness and elongation at the break of cassava starch films increased with increasing GEO concentration (from 0.5, 1, and 2%). However, increasing GEO concentration decreased the water content, water vapor permeability, and tensile strength of the prepared films'. Further, the addition of GEO increased the surface roughness, opacity, and antibacterial properties of the prepared films. With the increase of GEO concentration, L^*^ and a^*^ of cassava starch film decreased, while b^*^ and Δ E increased. This study provides a theory for cassava starch-based films as a biological packaging material.

## Introduction

Plastic films are storage methods that can protect food from contamination; however, the non-biodegrade plastics accumulate plastic wastes, resulting in environmental pollution (1). Presently, biodegradable films have become the focus of research. Biodegradable films can be degraded in the soil environment quickly, reducing, in turn, the accumulation of waste in the environment. Presently, the main biodegradable films are made from carbohydrate polymers, such as gelatin, chitosan, and starch (2–4). Therefore, biodegradable film packaging materials can reduce environmental pollution and natural resources (5).

Starch is widely used as the raw material for producing biodegradable films because of its rich resources, low price, and wide storage in various plants (6, 7). Starch films are an ideal biodegradable material with some advantages, such as being non-toxic, colorless, and tasteless (5). However, starch films have some shortcomings, including poor water resistance and mechanical properties (5). A series of shortcomings can be avoided by adding plasticizers to the starch film (5). Among all types of plant-derived starch, cassava starch is an excellent material for starch film production (8). It has many advantages, including low production cost and long drying time (8). The Food and Agriculture Organization of the United Nations recommends cassava starch as a critical source of food security (8).

Geranium essential oil (GEO) has a wide range of applications in the food industry. GEO has a specific therapeutic effect on eczema and beriberi disease (9). The main component of GEO is phenolics, and its antibacterial activity is closely related to phenols, especially flavonoids (10). On this occasion (11), found that GEO has a strong bacteriostatic effect on Gram-positive bacteria, such as *Staphylococcus epidermidis* and two strains of *Saccharomyces cerevisiae*.

GEO added to cassava starch-based films can achieve a sustained-release effect in food packaging (5, 12). Moreover, the addition of GEO could affect the films' physical and chemical properties, but none of the studies has dealt with this aspect. Hence, this study was carried out to (1) explore the effect of GEO on the physicochemical properties of cassava starch-based films and (2) evaluate the antibacterial activity of films incorporated with GEO.

## Materials and Methods

### Materials

Cassava starch, glycerin, and Tween 80 were procured from Beijing Solebo Company (Beijing, China). Geranium essential oil (GEO), Mueller-Hinton agar (MHA), and Mueller-Hinton broth (MHB) were acquired from Shanghai Yuanye Bio-Technology Co., Ltd. (Shanghai, China). Freeze-dried strains of *Staphylococcus aureus* (ATCC6538), *Escherichia coli* (ATCC25923), and *Listeria monocytogenes* (ATCC19115) were generously offered by the School of Food Science and Engineering, Qilu University of Technology (Jinan, China).

### Preparation of Films

Herein, cassava starch-based films were prepared by the casting method (13, 14). First, 6 g of cassava starch was weighed accurately and dissolved in 100 mL of deionized water. Afterwards, the starch suspension was stirred for 30 min in a magnetic agitator with a heating device (the parameters of the magnetic agitator were set to 800 rpm and 90°C). Then, 25% glycerol (based on the author's experience) was added to the film-forming solution. After that, different concentrations of GEO (0.5, 1, and 2%) were added to the film-forming solution (when the GEO was more than 3%, the film-forming solution underwent phase separation). The film-forming solution was stirred for 10 min in a magnetic agitator. The film-forming solution was poured into a special polytetrafluoroethylene (PTFF) mold (15 × 10 cm, Tianjin Kaitong Reagent Company, Tianjin, China). Next, the PTFF mold was placed in a 40°C oven and dried for 8 h. After drying, the cassava starch-based films were removed from the mold and maintained at relative humidity (RH) of 53% and 25°C for 96 h for further use.

### Thickness Measurement

The thickness of cassava starch-based films supplemented with different concentrations of GEO (control, 0.5, 1, and 2%) was measured using a spiral micrometer (No. 7301, Mitutoyo Co. Ltd., Tokyo, Japan) (15). There were four samples, and each sample was randomly selected at 5 different locations for measurement.

### Moisture Content

The moisture content (MC) of the prepared films was determined by the oven method (5). First, the quantity (M_1_) of cassava starch-based films was accurately weighed, then the sample was dried in an oven for 12 h at 110°C and weighed again (M_2_). The following formula was used to calculate the moisture content (%) of the prepared films:


$$\begin{array}{l} MC=\frac{M_{1}-M_{2}}{M_{1}}\;\times \text{100\%} \end{array}$$

Each sample was measured in triplicate, and the mean value was calculated.

### Solubility in Water

The solubility of the prepared films was measured as reported elsewhere (5, 16). First, the prepared films were cut into a square sample (2 × 2 cm), and the sample (M_3_) was accurately weighed. The prepared film sample was put into a conical bottle containing 100 mL of deionized water, and then the bottle was stirred at 180 rpm for 6 h. After stirring, the prepared film was oven-dried at 110°C for 7 h, and the film sample mass (M_4_) was weighed. The solubility of the prepared films was calculated according to the following formula:


$$\begin{array}{l} S=\frac{M_{3}-M_{4}}{M_{3}}\times \text{100} \end{array}$$

Each sample was measured in triplicate, and the mean value was calculated.

### Water Vapor Permeability

Herein, the water vapor permeability (WVP) of cassava starch-based films was measured by a water vapor permeation meter (PERME™ W3/030, Lab think Instruments Co., Ltd., Jinan, China) (15). Before the test, the prepared films were maintained at 53% RH and 25°C for 96 h. Then, the prepared films were made into a round sample of 33 cm^2^. Afterwards, the sample was measured in a water vapor permeability meter (90% RH and 30°C) for 12 h. The WVP of cassava starch-based films was calculated from three repeated specimens.

### Film Color Measurement

The color of cassava starch-based films was determined by a colorimeter (ADCI-60-C, Beijing, China) (5, 17). The parameters of the colorimeter are as follows: the light source/angle is D65/10°, and the opening is 30 mm. First, the sample was placed on a standard whiteboard to determine the parameters L${}_{0}^{*}$ (luminance index), a${}_{0}^{*}$ (hue from red to green), b${}_{0}^{*}$ (hue from yellow to blue), and total color difference (ΔE). Then, the colorimeter was placed on the prepared films for measurement. Each prepared film sample was randomly measured at five points, and the average value was calculated (L${}_{0}^{*}$ = 91.69, a${}_{0}^{*}$ = 8.30, b${}_{0}^{*}$ = 4.32).


$$\begin{array}{l} \Delta E=\sqrt{{\left({L_{0}}^{*}-L^{*}\right)}^{2}+{\left({a_{0}}^{*}-a^{*}\right)}^{2}+{\left({b_{0}}^{*}-b^{{}^{*}}\right)}^{2}}\; \end{array}$$

Where L${}_{0}^{*}$, a${}_{0}^{*}$ and b^*^ are the color parameters of a white standard.

### Mechanical Properties

The mechanical properties of the prepared films were tested as described by others (18–20). First, the prepared films were cut into a rectangular shape of 10 × 15 cm. Then, the samples were put into an automatic tensile testing machine (Param Xlw, Jinan, China) to determine the tensile strength (TS, MPa) and elongation at break (EAB, %). The tensile speed of the automatic tensile testing machine is set to 100 mm/min, and the values of TS and EAB are read after the test is completed. Each film sample was repeated at least six times.

### Antibacterial Activity of the Prepared Films

In this experiment, the antibacterial activity of cassava starch-based films supplemented with GEO was determined by the disk diffusion method (5, 14, 21, 22). First, the prepared films were cut into a disc shape with a diameter of 6 mm under aseptic conditions. Then, the samples were placed in a Petri dish, in which an overnight culture of 100 μL had been seeded beforehand. Each Petri dish contained ~10^8^ CFU/mL (0.5 McFarland) of *Staphylococcus aureus, Escherichia coli*, and *Listeria monocytogenes*. The dish was incubated at 37°C for 24 h, and the area of the zone of inhibition was measured with a digital caliper (Mitutoyo No. 192-30, Tokyo, Japan). Chloramphenicol was used as a reference standard (23).

### Atomic Force Microscopy

Atomic force microscopy (AFM) was used to observe the surface of the prepared films (5). Before the experiment, the prepared film sample was first placed in an environment of 53% RH and 25°C for 48 h. The AFM adopts tap mode, and the observation ranges are 5 × 5 and 10 × 10 μm. In addition, the following parameters related to sample roughness are calculated: average roughness (Ra: the arithmetic average of the absolute values of the profile height deviations from the mean line) and root mean square roughness (Rq: root mean square average of height deviations taken from the mean data).

### Statistical Analyses

SPSS software (Version 13.0, Statistical Package for the Social Sciences Inc., Chicago, USA) was used to analyze the mean ± standard deviation of each experimental data. A one-way analysis of variance and Duncan's multiple range tests were performed. *p* < 0.05 was considered statistically significant.

## Results and Discussion

### Thickness

As shown in Table 1, the thickness of the prepared films is between 0.16 ± 0.008 and 0.22 ± 0.009 mm. From Table 1, it is apparent that the thickness of cassava starch-based films fortified with GEO increased compared with the control (unmodified) film, indicating that the thickness of the prepared films can be increased by adding GEO. In this context (24), stated that the inclusion of essential oil significantly increased the thickness of the cornstarch-based films compared with the unmodified starch-based films. Herein, we found that the thickness of the prepared films increased from 0.18 ± 0.005 to 0.22 ± 0.009 mm, and the thickness increased by 22% compared with the control film. This finding denotes that the thickness of the prepared films increased with increasing GEO concentration.

**Table 1:** Functional properties of the cassava starch-based films supplemented with GEO.

**Film type**	**Thickness (mm)**	**Moisture content (MC) (%)**	**Solubility in water (%)**	**Water vapor permeability** **[g·cm/(cm2·s·Pa)]**
Control	0.16 ± 0.008^a^	17.23 ± 0.64^a^	32.47 ± 0.19^a^	3.53 ± 0.20^a^
GEO (0.5%)	0.18 ± 0.005^ab^	18.16 ± 0.03^ab^	33.43 ± 0.07^b^	3.11 ± 0.14^a^
GEO (1%)	0.19 ± 0.007^b^	18.40 ± 0.10^b^	34.33 ± 0.15^c^	2.51 ± 0.08^b^
GEO (2%)	0.22 ± 0.009^c^	18.53 ± 0.14^b^	37.63 ± 0.32^d^	2.03 ± 0.17^b^

*Data are presented as the X ± SD, and different superscript letters in the same column denote significant differences (Duncan's range test, p < 0.05)*.

### Mechanical Properties

The mechanical properties of the prepared films are important indexes to judge whether they are used as packaging materials. Mechanical properties can be divided into tensile strength (TS) and elongation at break (EAB). TS can reflect strength, and EAB reflects the flexibility of cassava starch-based films. Table 2 shows the TS and EAB of the prepared films with and without GEO inclusion. As shown in Table 2, the TS of cassava starch-based films with GEO inclusion decreases while the EAB increases continuously (TS: 8.12 ± 0.30–3.09 ± 0.11 MPa, EAB: 41.59 ± 1.67–72.84 ± 3.55 %). The mechanical properties of the prepared films can be changed with the inclusion of GEO. With increasing GEO concentration, the TS of the prepared films decreased, and the EAB increased. The reason may be that the addition of GEO leads to the formation of a discontinuous matrix in the prepared films, which tends to decrease the internal force between polymers, ultimately decreasing the TS and increasing the EAB of the prepared films (25). Other studies have shown that the change in the mechanical properties of starch-based films might be attributed to the interactions between polar polymers and non-polar lipid molecules (26). Further, it was shown that the preparation method of starch films, sample thickness, test methods, and other factors also could affect the mechanical properties of starch-based films (12).

**Table 2:** Tensile strength and elongation at break of cassava starch-based films incorporated with geranium essential oil (GEO).

**Sample**	**Control**	**0.5%**	**1%**	**2%**
TS	8.12 ± 0.30^a^	6.15 ± 0.30^b^	5.04 ± 0.22^c^	3.09 ± 0.11^d^
EAB	41.59 ± 1.67^a^	47.97 ± 3.36^a^	59.44 ± 4.51^b^	72.84 ± 3.55^c^

*Data are shown as the X ± SD, and different superscript letters in the same row indicate significant differences (Duncan's range test, p < 0.05)*.

### Solubility in Water

Solubility is a crucial property of food packaging materials (24). Different solubility food packaging materials are selected according to the needs of the food itself (24). When packaging food with high moisture content, it is necessary to use materials with lower solubility; in contrast, food packaging materials with higher solubility are needed. As shown in Table 1, the solubility of cassava starch-based films containing GEO gradually increased compared with the unmodified films (32.47 ± 0.19–37.63 ± 0.32%). With the increase in GEO concentration, the solubility of the prepared films gradually increased, which may be due to the rupture of cassava starch-based films during the experiment, which reduced the insertion of water into the prepared films (15). Other research studies have declared that due to the increased thickness and surface roughness of starch-based films with essential oil, the contact area between starch-based films and water increases, which increases the solubility of starch-based films (15). Cassava starch-based films containing GEO have high solubility and can be used to package food with low moisture content to ensure a low moisture environment (22).

### Moisture Content

The moisture content of starch-based films is closely related to the incorporated substances. If hydrophilic substances are added then the moisture content of starch-based films increases, and if the added material is hydrophobic, the moisture content of the films decreases (27). Herein, the experimental sample is a cassava starch film containing GEO, a hydrophobic substance that can theoretically reduce the moisture content of cassava starch. However, Table 1 shows that the solubility of cassava starch increases with increasing GEO (17.23 ± 0.64–18.53 ± 0.14%). The reason may be that GEO forms a porous structure in cassava starch-based films, leading to the increased moisture content in the prepared films. Another study has found that the increase in moisture content in starch-based films containing essential oil may be related to the rupture of starch films (28). According to these studies and theories, the inclusion of hydrophobic GEO may increase the moisture content of cassava starch-based films.

### Atomic Force Microscopy

**Figure 1:**
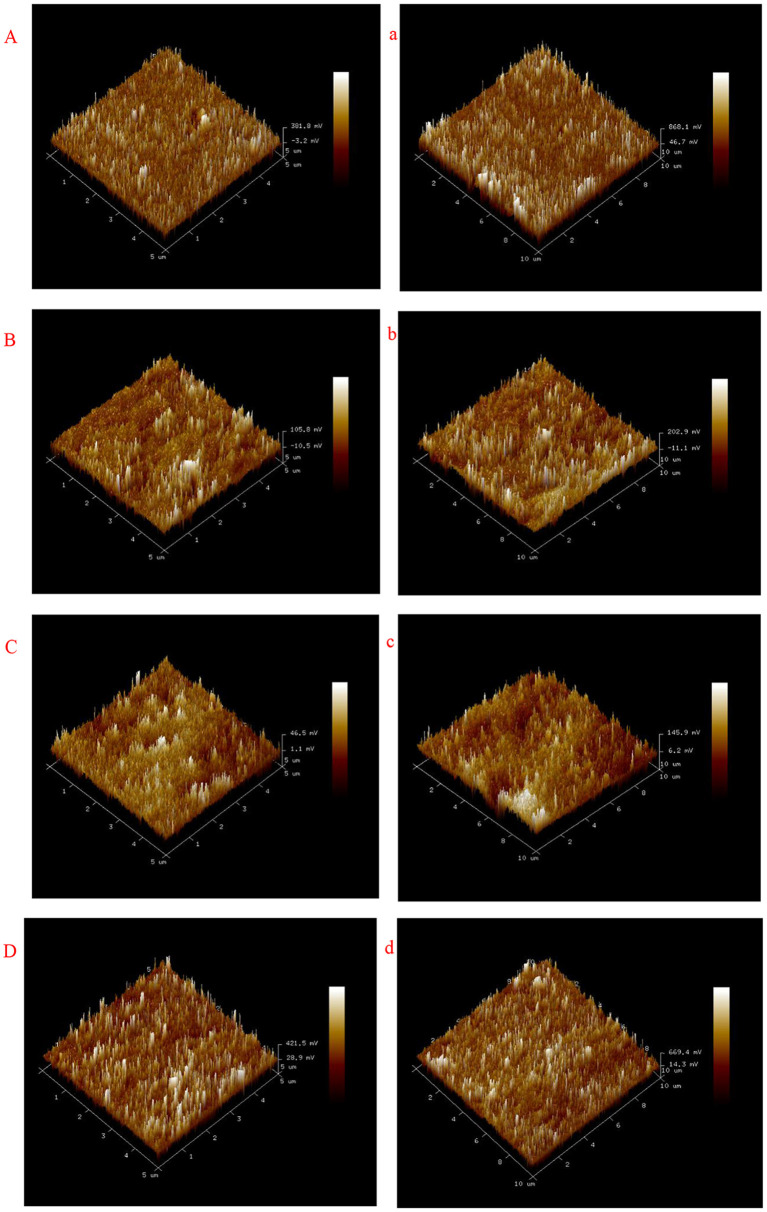
Typical atomic force microscopy (AFM) images of cassava starch-based films (5 and 10 μm): **(A)** Control (5 μm), **(B)** 0.5% (5 μm), **(C)** 1% (5 μm), and **(D)** 2% (5 μm), **(a)** Control (10 μm), **(b)** 0.5% (10 μm), **(c)** 1% (10 μm), and **(d)** 2% (10 μm).

Atomic force microscopy (AFM) is commonly used to image the surface roughness of biopolymers at the nanometer scale. In our study, we used atomic force microscopy to observe the surface of starch-based films (5, 24). We used tapping mode to observe cassava starch-based films with and without GEO inclusion. Figure 1 and Table 3 show that the Rq and Ra values of cassava starch-based films with added GEO increase in the scanning ranges of 5 × 5 and 10 × 10 μm, indicating that the surface roughness of the prepared films can be improved by adding GEO. Cassava starch-based film roughness index (Rq and Ra) increased gradually with increasing GEO concentration. We may imply that the cassava starch-based films with the addition of GEO agglomerates in film drying lead to an increase in the roughness of the cassava starch films (29) concluded that the roughness of starch-based films with essential oil is larger than the control films (16) also obtained similar results in their study in which the inclusion of oleic acid beeswax increased the surface roughness. This roughness may be related to oil droplets and the surface irregularity caused by the accumulation of oil droplets during drying.

**Table 3:** AFM roughness parameters of the cassava starch-based films.

**Scanning range**	**0%**	**0.5%**	**1%**	**2% GEO**
	**GEO**	**GEO**	**GEO**	**GEO**
5 μm^2^ Rq	34.2	36.1	36.6	50.5
5 μm^2^ Ra	26.9	29.2	38.9	49.1
10 μm^2^ Rq	80.1	100.3	111.5	129.6
10 μm^2^ Ra	57.9	79.5	85.8	108.0

### Microbiological Activity

Table 4 lists the antibacterial activities of cassava starch-based films incorporated with different concentrations of GEO. The tested strains were *Escherichia coli, Listeria monocytogenes*, and *Staphylococcus aureus*. The antibacterial properties of different cassava starch-based films were compared based on the area of inhibition. It is shown that the prepared films with GEO have stronger antibacterial properties than films without GEO (*E. coli*: 0–1236.16 ± 11.61, *S. aureus*: 0–763.44 ± 10.47, *L. monocytogenes*: 0–963.91 ±11.12 mm^2^). Therefore, the prepared films can be used as a suitable geranium oil carrier. When cassava starch-based films contact agar, they can slowly release GEO, thus displaying an antibacterial role. Using starch-based films as carriers to slowly release essential oil for antibacterial activity can have a long-term bacteriostatic effect to ensure the nutrition and quality of food (26, 30).

**Table 4:** Antibacterial activities of the cassava starch-based film against Gram-negative and Gram-positive bacteria.

**Film type**	**Inhibition zone (mm** ^**2**^ **)**		
	***E. coli***	***S. aureus***	***L. monocytogenes***
Control	0^a^	0^a^	0^a^
GEO (0.5%)	58.37 ± 2.13^b^	69.35 ± 1.35^b^	71.85 ± 3.47^b^
GEO (1%)	69.21 ± 2.44^c^	93.45 ± 3.34^c^	98.64 ± 3.21^c^
GEO (2%)	99.14 ± 3.21^d^	125.31 ± 2.58^d^	126.57 ± 2.45^d^
Chloramphenicol	1236.16 ± 11.61^e^	763.44 ± 10.47^e^	963.91 ± 11.12^e^

*Data are shown as the X ± SD, and different superscript letters in the same column indicate significant differences (Duncan's range test, p < 0.05)*.

As shown in Table 4, the bacteriostatic effect on Gram-positive bacteria (*Staphylococcus aureus* and *Listeria monocytogenes*) was greater than that of Gram-negative bacteria (*E. coli*) (31) reported that Gram-positive bacteria have a layer of peptidoglycan and that peptidoglycan has a specific blocking effect limiting the diffusion of hydrophobic compounds (essential oils), resulting in the poor bacteriostatic effect. The essential oil itself has a specific bacteriostatic effect and has been widely used in the food processing industry. As shown in Table 4, with an increase in GEO concentration, the antibacterial effect of cassava starch-based films becomes increasingly stronger. The essential oil can destroy the cell membrane and further deplete the cell's contents to achieve a bacteriostatic effect. In this context (31), declared that starch films with essential oil have a specific bacteriostatic effect and can be used as bacteriostatic packaging materials.

### Optical Properties

Optical properties are important indicators of packaging materials. Customers can choose packaging materials with different optical properties according to their properties. Table 5 lists the optical indexes (L, a, and b) of cassava starch-based films. With increasing GEO concentration, the brightness of cassava starch films (L) decreases continuously (66.17 ± 2.35–60.78 ± 1.18). GEO can affect the brightness (L) of the prepared films. The reason may be that the addition of GEO leads to a change in light refraction on the surface of the prepared films, which leads to a decrease in brightness. Diffusion of essential oil in starch films leads to a change in the optical properties of cassava starch-based film, as reported by (24, 32). Further, with the increase in GEO concentration, the redness index (a) of the prepared films decreases (7.94 ± 0.68–6.22 ± 0.47), and the yellowness index (b) increases continuously (2.43 ± 0.21–3.69 ± 0.54). The reason may be that the color of GEO itself is mixed with cassava starch-based films, which leads to a change in the prepared films (32). This experiment proves that cassava starch-based films with GEO inclusion can be used as a food packaging material to avoid light preservation. With the increase of GEO concentration, the ΔE of cassava starch film increased gradually with significant differences compared with the unmodified films (*p* < 0.05). The results showed that GEO significantly affected the ΔE of cassava starch film (5) declared that with the continuous increment of the *Zanthoxylum bungeanum* essential oil concentration, the ΔE of corn starch film gradually increases with a significant difference.

**Table 5:** Color parameters (L*, a*, b*) of cassava starch-based films.

**Film type**	L*	a*	b*	**ΔE**
Control	66.17 ± 2.35^a^	7.94 ± 0.68^a^	2.43 ± 0.21^c^	0.76 ± 0.05^c^
GEO (0.5%)	63.25 ± 2.14^b^	7.32 ± 0.53^b^	3.00 ± 0.15^b^	2.11 ± 0.06^b^
GEO (1%)	61.03 ± 2.36^c^	6.36 ± 0.21^c^	3.43 ± 0.56^a^	2.18 ± 0.13^b^
GEO (2%)	60.78 ± 1.18^c^	6.22 ± 0.47^c^	3.69 ± 0.54^a^	5.16 ± 0.35^a^

*Data are shown as the X ± SD, and different superscript letters in the same column indicate significant differences (Duncan's range test, p < 0.05)*.

### Water Vapor Permeability

Water vapor permeability (WVP) is one of the critical factors affecting the quality of food packaging materials. The primary function of food packaging is to avoid or reduce the water transfer between food and surrounding media or between two components of different types of food. With the increase in GEO concentration, the water vapor rate of cassava starch film decreased [3.53 ± 0.20–2.03 ± 0.17 g·cm/(cm2·s·Pa)]. The reason is that with the increase of GEO concentration, amylose and oil in cassava starch form a lipid complex (V-shaped crystallization), which increases the compactness of cassava starch film, leading to a decrease in water vapor permeability (33) stated that oil could form lipid complex with amylose, thus improving the permeability of biofilms.

## Conclusions

In this study, cassava starch-based films were prepared by the casting method. The effects of different concentrations of GEO (0.5, 1, and 2%) on the physicochemical and antibacterial properties were assessed. The tensile strength of cassava starch-based films with GEO was lower than that of the unmodified films. However, the elongation at break was higher than that of the control films. The addition of GEO changed the flexibility of cassava starch-based films. The inclusion of GEO changed the water vapor transmittance and optical properties of the prepared films. With increasing GEO concentration, the roughness of cassava starch-based films increased gradually. In addition, films supplemented with GEO showed good antibacterial activity, and its bacteriostatic effect on Gram-positive bacteria was higher than that of Gram-negative bacteria. With the increase of GEO concentration, L^*^ and a^*^ of cassava starch film decreased, while b^*^ and ΔE increased. This study revealed that cassava starch-based films with GEO have good physicochemical properties and antibacterial activity, which effectively prepare functional packaging materials.

## Data Availability Statement

The raw data supporting the conclusions of this article will be made available by the authors, without undue reservation.

## Author Contributions

BW: investigation, software, visualization, and writing—original draft. SY and LQ: supervision and project administration. WG, XK, BY, and PL: formal analysis. BC: conceptualization, methodology, writing—review and editing, and supervision. AMA: conceptualization, methodology, writing—review and editing, and supervision. All authors contributed to the article and approved the submitted version.

## Funding

This project was funded by the National Key Research & Development Program in China (2019YFD1002704), Key Research and Development Program of Shandong Province (2021CXGC010808, 2021CXGC010807, and 2019JZZY010722), the Innovation Team of Jinan City (2018GXRC004), Special Funds for Taishan Scholars Project (NO. ts201712060), Special Project of International Cooperative Research (QLUTGJH2018016), Shandong Bohai Sea Granary Science and Technology Demonstration Project (2019BHLC002), and Innovation Pilot Project of Integration of Science, Education and Industry of Shandong Province (2020KJC-ZD011).

## Conflict of Interest

LQ was employed by Zhucheng Xingmao Corn Developing Co., Ltd. The remaining authors declare that the research was conducted in the absence of any commercial or financial relationships that could be construed as a potential conflict of interest.

## Publisher's Note

All claims expressed in this article are solely those of the authors and do not necessarily represent those of their affiliated organizations, or those of the publisher, the editors and the reviewers. Any product that may be evaluated in this article, or claim that may be made by its manufacturer, is not guaranteed or endorsed by the publisher.
